# Frustrated Magnetism in FeGe_3_O_4_ with a Chiral Trillium Network

**DOI:** 10.1021/jacs.5c22025

**Published:** 2026-02-12

**Authors:** Matt Boswell, Mingyu Xu, Haozhe Wang, Mouyang Cheng, Na Li, Xuefeng Sun, Haidong Zhou, Huibo Cao, Mingda Li, Weiwei Xie

**Affiliations:** † Department of Chemistry, 3078 Michigan State University, East Lansing, Michigan 48824, United States; ‡ Quantum Measurement Group, 2167 Massachusetts Institute of Technology, Cambridge, Massachusetts 02139, United States; § Department of Materials Science and Engineering, Massachusetts Institute of Technology, Cambridge, Massachusetts 02139, United States; ∥ Center for Computational Science & Engineering, Massachusetts Institute of Technology, Cambridge, Massachusetts 02139, United States; ⊥ Anhui Provincial Key Laboratory of Magnetic Functional Materials and Device, Institutes of Physical Science and Information Technology, 12487 Anhui University, Hefei, Anhui 230601, China; # Department of Physics and Astronomy, University of Tennessee, Knoxville, Tennessee 37996, United States; ∇ Neutron Scattering Division, 6146 Oak Ridge National Laboratory, Oak Ridge, Tennessee 37831, United States; ○ Department of Nuclear Science and Engineering, Massachusetts Institute of Technology, Cambridge, Massachusetts 02139, United States

## Abstract

The discovery of new magnetic ground states in geometrically frustrated lattices remains a central challenge in materials science. Here, we report the synthesis, structural characterization, and frustrated magnetic properties of FeGe_3_O_4_, a newly identified compound that crystallizes in the noncentrosymmetric cubic space group *P*2_1_3. In this structure, Fe atoms form an intricate double-trillium lattice with nearest-neighbor Fe–Fe distances of ∼4.2 Å, while Ge^2+^ ions mediate magnetic interactions through Fe–Ge–Fe pathways. Field-dependent magnetization at 2 K shows a pronounced nonlinearity, reaching a maximum moment of 2.55(3) μ_B_/Fe^2+^ at 70 kOe without evidence of saturation. Magnetic susceptibility, heat capacity, and neutron scattering collectively reveal the onset of short-range magnetic interactions near 5 K, with no long-range ordering detected down to 0.06 K. Specific heat measurements demonstrate strong frustration: only ∼34% of the expected magnetic entropy is recovered at 2.4 K. Taken together, these results establish FeGe_3_O_4_ as a rare example of a geometrically frustrated trillium lattice magnet, offering a promising platform for exploring exotic quantum magnetic phenomena.

## Graphical abstract

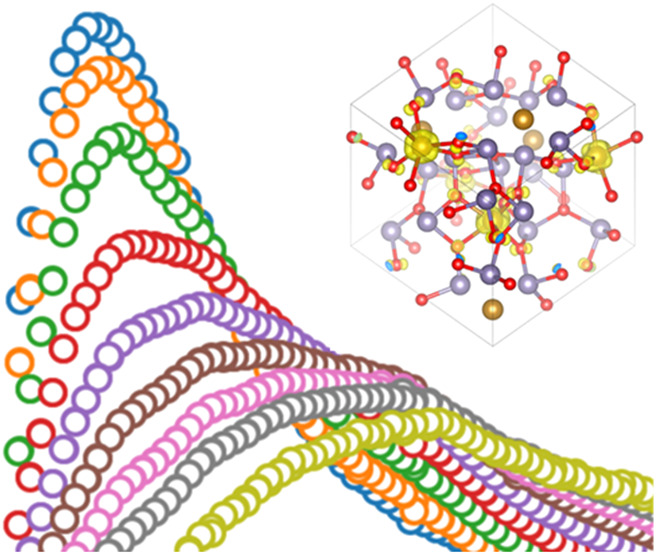

## Introduction

Geometrically frustrated magnets are of significant interest due to their macroscopic ground-state degeneracies, which give rise to a variety of exotic quantum effects.^1–3^ A common feature in the interaction networks of frustrated materials is the presence of odd-membered rings, such as triangles and pentagons.^4^ Despite the diverse structural topologies that can give rise to frustration, much of the research in this field has focused on a relatively limited subset of structure types commonly found in oxide materials, including pyrochlore, triangular, Kagome, and face-centered cubic lattices.^5–7^ Expanding the study of geometric frustration to a less common lattice geometry offers the potential to uncover novel magnetic phases and their associated physics. Recently, the cubic trillium lattice, characterized by the chiral space group *P*2_1_3, has been investigated as a framework for frustrated magnetism. This lattice features a network of corner-sharing triangles, which naturally predisposes it to geometric frustration.^8–12^ Although magnets adopting the trillium lattice are rare, primarily intermetallics referred to as B20 compounds, they exhibit a wide array of intriguing physical phenomena.^13^ For instance, in MnSi and CoSi, magnetic skyrmions emerge due to the interplay between ferromagnetism and the Dzyaloshinskii-Moriya interaction.^14,15^ FeSi has been proposed as a d-electron topological Kondo insulator candidate.^16^ Other compounds, such as EuPtSi and EuPtGe, display behavior consistent with strongly correlated spin liquids,^17,18^ while CeIrSi is considered a candidate for Ising trillium spin ice.^19^ Theoretically, a simple nearest-neighbor Heisenberg antiferromagnet on the chiral trillium lattice has been predicted to support a classical spin liquid (CSL) phase over a wide temperature range, with 120° helical order in its frustrated magnetic ground state.^11,20^ From a structural perspective, B20 compounds such as MSi (M = Mn, Fe, Co) contain a single trillium lattice with nearest M-M distances around 2.7 Å. These short distances often promote long-range magnetic ordering, even under conditions of geometric frustration.^21^ For instance, external stimuli, such as pressure-induced quantum phase transitions, can drive emergent behaviors in these systems.^22,23^ However, theoretical studies suggest that the degree of frustration in single trillium lattices like those in B20 compounds is insufficient to suppress long-range order entirely due to the short M-M distance.

Later investigations, both experimental and computational, have revealed that quantum spin liquid (QSL) behavior can arise in systems with interconnected trillium lattices. Representative examples include compounds in the langbeinite family, K_2_M_2_(SO_4_)_3_ (M = Fe, Co, Mn, Cr), where the interconnected trillium lattices feature nearest M-M distances ranging from 4.4 to 6.2 Å.^24,25^ Another notable system, NaMn­(HCOO)_3_, contains magnetic Mn^2+^ (*S* = 5/2) ions arranged in a trillium lattice with nearest-neighbor Mn–Mn distances of approximately 5.6 Å.^26^ The compound K_2_M_2_(SO_4_)_3_ features a network of trigonally distorted MO_6_ octahedra, which are interconnected via SO_4_^2–^ groups. This connectivity establishes an M–O–S–O–M supersuperexchange pathway that mediates magnetic interactions between M^2+^ ions. Within this structure, there are two distinct crystallographic M^2+^ sites, differentiated by their M-O bond distances, with each site forming a single trillium lattice. Similarly, in NaMn­(HCOO)_3_, neighboring Mn^2+^ cations are connected by HCOO^–^ ions, resulting in an Mn–O–C–O–Mn superexchange pathway. Both compounds exhibit characteristics of geometric frustration, including the suppression of long-range magnetic order and the emergence of spin-liquid-like behavior, driven by the intricate interplay of their structural and magnetic interactions.

To the best of our knowledge, no compound has been reported to adopt a chiral trillium network with significantly larger M-M distances that would effectively suppress long-range magnetic interactions via direct exchange. Moreover, such a system featuring an M-O-M or M-T-M pathway for magnetic superexchange has not yet been observed. The lack of compounds with these characteristics represents a critical gap in the exploration of geometric frustration and the potential emergence of novel magnetic phases in trillium lattice framework. On the other hand, Fe and Ge form various binary phases, including the B20 FeGe phase.^27^ Based on this, our research focused on Fe–Ge–O ternary phases to investigate the potential for discovering novel trillium phases. Herein, we present a comprehensive structural characterization along with an analysis of the magnetic frustration of FeGe_3_O_4_, emphasizing the Fe–Fe exchange interactions within the trillium lattice.

## Experiments and Calculations

### Synthesis of FeGe_3_O_4_ Crystals

FeGe_3_O_4_ crystals were synthesized by using the chemical vapor transport (CVT) method. Stoichiometric amounts of Fe granules (Alfa Aesar, 99.98%), Fe_2_O_3_ powder (JT Baker, Baker Analyzed Reagent grade), finely ground Ge pieces (Thermo Scientific, 99.9999+%), and GeO_2_ powder (Alfa Aesar, 99.999%) were mixed thoroughly in an atomic ratio of 2:2:9:9, with a total mass of approximately 300 mg. To facilitate the transport process, ∼50 mg I_2_ flakes (Fisher Chemical) were added as the chemical transport agent. The mixture was sealed in a quartz tube under vacuum (∼10^–5^ Torr) and subjected to a thermal gradient by heating to 600 °C for 1 week. Subsequently, the tubes were cooled to room temperature at a controlled rate of 10 °C per hour. As a result, red transparent crystals with dimensions of approximately 1–2 mm were successfully grown. All products are stable toward decomposition in air and moisture.

### Phase Analyses and Chemical Compositions

The synthesized samples were finely ground and analyzed for phase identification and purity using powder X-ray diffraction (PXRD) on a Bruker Davinci powder X-ray diffractometer equipped with Cu Kα radiation (λ_Kα_ = 1.5406 Å). The upper and lower discriminator values were set to 0.40 and 0.18 V, respectively, to mitigate the background due to fluorescence. Diffraction patterns were collected over a 2θ range of 5–120° with a step size of 0.010° in step-scan mode, utilizing a scintillation detector. Phase identification and lattice parameter determination were performed through Rietveld refinements using the GSAS-II software package.^28^ Chemical composition analysis was conducted using a JEOL 6610LV scanning electron microscope (SEM) coupled with an energy-dispersive X-ray spectroscopy (EDS) detector (Oxford Instruments Isis X-ray analyzer). Samples were affixed to carbon tape before placement in the SEM chamber and analyzed at an accelerating voltage of 20 kV. Spectra were acquired with a collection time of 100 s, examining multiple points within each phase across various grains. Compositional estimates were refined by Oxford’s SEM Quant software, incorporating corrections for matrix effects to ensure accuracy.

### Crystal Structure Determination

A single crystal with dimensions of 0.098 × 0.063 × 0.029 mm^3^ was picked up, mounted on a nylon loop with paratone oil, and measured using a Rigaku XtalLAB Synergy, Dualflex, Hypix single-crystal X-ray diffractometer equipped with an Oxford Cryosystems 800 low-temperature device. Data acquisition was performed using ω scans with Mo K_α_ radiation (λ = 0.71073 Å, microfocus sealed X-ray tube, 50 kV, 1 mA). The measurement strategy, including the total number of runs and images, was determined using the strategy calculation feature in CrysAlisPro software (version 1.171.43.143a, Rigaku OD, 2024). Data reduction induced correction for Lorentz polarization. Numerical absorption correction based on Gaussian integration over a multifaceted crystal model. Empirical absorption correction was applied using spherical harmonics implemented in SCALE3 ABSPACK scaling algorithm. Structure solutions and refinement were conducted using SHELXTL Software Package.^29^

### Physical Properties Measurements of FeGe_3_O_4_

Temperature- and magnetic-field-dependent magnetization measurements of FeGe_3_O_4_ single crystals were carried out using a Quantum Design Magnetic Property Measurement System (MPMS3). The direct-current magnetic susceptibility measurements were performed in the temperature range of 2–300 K under both zero-field-cooled (ZFC) and field-cooled (FC) modes, with an applied field of 100 Oe and 1 kOe. Field-dependent isothermal magnetization measurements were also performed employing magnetic fields ranging from 0 to 7 T and at various temperatures. Temperature-dependent specific heat measurements on the crystals ranging in mass from 1 to 3 mg were carried out using a Quantum Design, Physical Property Measurement System (PPMS DynaCool) in the temperature range of 2.4–100 K at various applied magnetic fields up to 9 T. Low-temperature specific heat measurements on the crystals with the total mass ∼6.4 mg were carried out down to 0.06 K without an applied magnetic field.

### Single-Crystal Neutron Diffraction

Single-crystal neutron diffraction was conducted at CORELLI at the Spallation Neutron Source at Oak Ridge National Laboratory. Two crystals were measured, both at 2 and 20 K with angles ranging from 0 to 360° incrementing by 3°.^200^

## Results and Discussion

### Synthesis and Structural Analysis of FeGe_3_O_4_

Synthetic attempts to prepare FeGe_3_O_4_ by reacting Fe_2_O_3_, GeO_2_, and Ge as a reducing agent resulted in mixtures of FeGe_3_O_4_ and FeGeO_3_ phases, as predicted by the phase diagram in Figure S1. To gain deeper insight into the structural features of FeGe_3_O_4_, single-crystal X-ray diffraction analysis was performed, focusing on elemental distributions, interatomic distances, and coordination environments, as shown in Figure 1a–d. The results of single-crystal diffraction are detailed in Tables S1 and S2. FeGe_3_O_4_ crystallizes in the noncentrosymmetric cubic space group *P*2_1_3 (No. 198), with two distinct Fe sites occupying the 4a Wyckoff positions. Although Fe^2+^ and Ge^2+^ were initially considered as mixed occupancies at these sites, refinement indicated no observable site mixing. The Fe atoms in the 4a sites form a unique double-trillium lattice. This interconnected lattice structure exhibits nearest Fe–Fe distances of approximately 4.2 Å.

**Figure 1:**
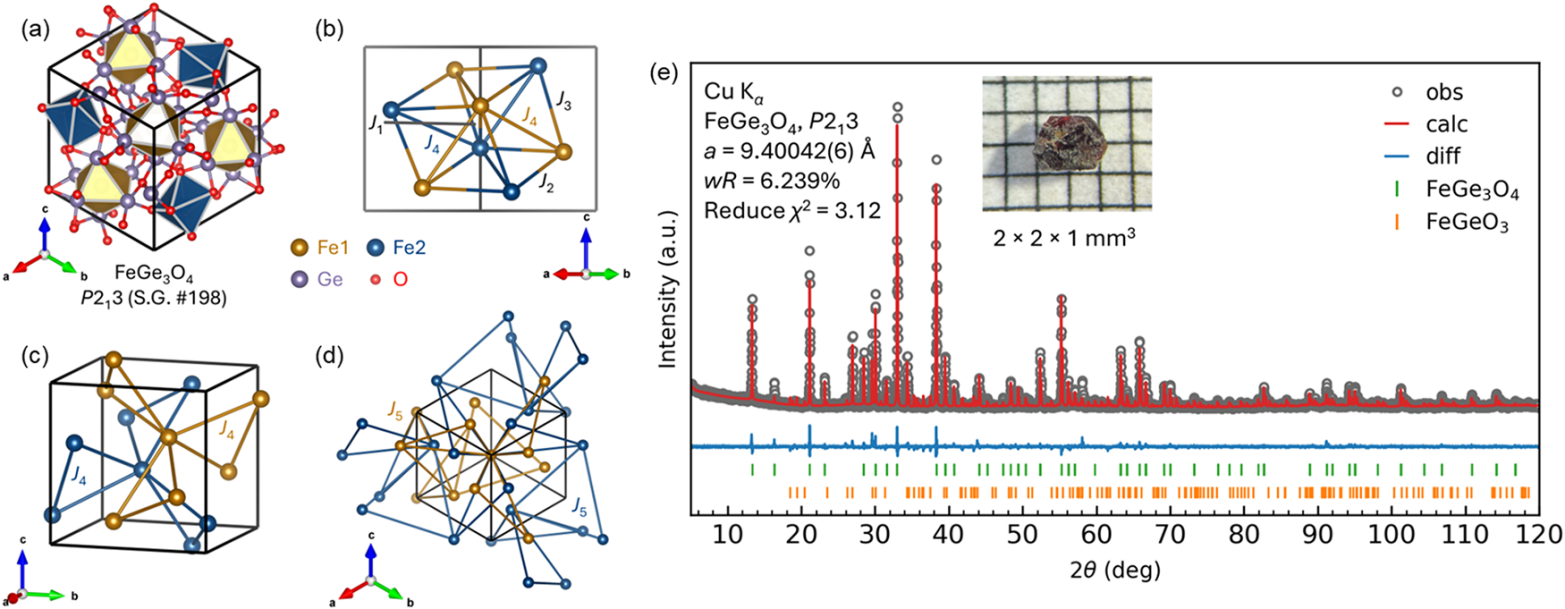
(a) Crystal structure of FeGe_3_O_4_ (*P*2_1_3, S.G. 198) with two distinct [FeGe_6_] and [FeO_6_] polyhedra shown. Brown, blue, violet, and red represent Fe1, Fe2, Ge, and O atoms. (b–d) Exchange interactions (*J*_1_ to *J*_5_ between Fe^2+^ ions). (e) Powder XRD pattern and Rietveld refinement of FeGe_3_O_4_. Bragg peak positions of each phase included are represented by vertical tick marks. FeGeO_3_ exists as an impurity powder. (Inset) Optical microscope image (1 mm graph paper) of FeGe_3_O_4_.

For the powder X-ray diffraction (PXRD) patterns, all scale factors and lattice parameters were refined, resulting in a reduced χ^2^ value of approximately 3.12 and weighted profile residuals (*R*_wp_) of 6.2%. The diffraction pattern shown in Figure 1e confirms that FeGe_3_O_4_ is the predominant phase in the synthesized products, with a minor secondary phase of FeGeO_3_ present at an estimated concentration of less than 5%. Consistent with single-crystal diffraction and SEM analyses, all refinements confirm a Fe/Ge molar ratio close to 1:3 for this phase. This agreement across multiple characterization techniques validates the structural and compositional integrity of the synthesized FeGe_3_O_4_ sample. Single crystals of FeGe_3_O_4_ were manually selected for physical property measurements to ensure the sample quality and phase purity.

The temperature dependence of magnetization in both ZFC and FC modes is presented in Figure 2a with no significant difference. The temperature-dependent magnetization measurement shows tail-like behavior without any feature indicating a phase transition. As shown in the inset of Figure 2a, magnetization at 1 kOe is fitted using Curie–Weiss (CW) law (χ = *C*/(*T* – θ) + χ_0_) from 60 to 300 K. Here, χ represents the magnetic susceptibility, C is the Curie constant, *T* is the temperature, θ is the Weiss temperature, and χ_0_ is a temperature-independent susceptibility term. The effective moment is around 4.81 μ_B_, which suggests that the Fe ion’s valence is +2 and is at a high-spin state (high-spin Fe^2+^ μ_eff_ = 4.9 μ_B_). This result consists of the hypothesis of the FeGe_3_O_4_ valence states in the phase diagram shown in Figure S1. Also, the Weiss temperature is negative (−2 K), which indicates dominant antiferromagnetic interactions in the FeGe_3_O_4_, and is similar to the one observed in another quantum spin liquid candidate YbMgGaO_4_ (θ = −4 K).^30^

**Figure 2:**
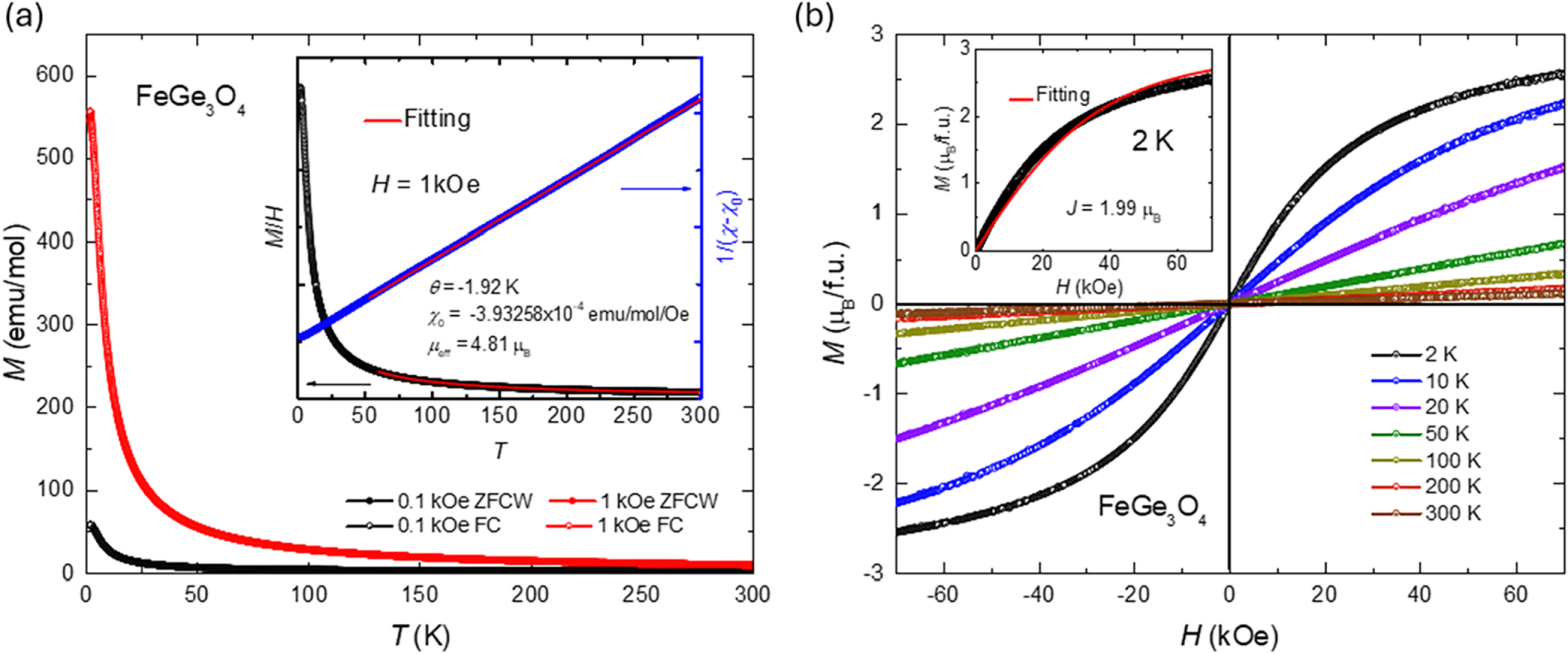
(a) Temperature dependence of magnetic susceptibility at 0.1 and 1 kOe. Inset: Curie–Weiss fitting result. (b) Magnetic field dependence of magnetization with the Brillouin function fitting.

As shown in Figure 2b, field-dependent magnetization measurements show nonlinear behavior at 2 K. The observed maximum magnetization, *M*_max_ = 2.55(3) μ_B_/Fe, was not saturated when the maximum field of 70 kOe was applied. Magnetization curves measured at various temperatures exhibit field-dependent behavior that can be fitted using the Brillouin function as follows^31^

$$M=M_{s}B_{J}\left(\frac{g_{J}\mu_{B}JB}{k_{B}T}\right)$$

$$M_{s}=g_{J}J$$

$$g_{J}=\frac{3J\left(J+1\right)+S\left(S+1\right)-L\left(L+1\right)}{2J\left(J+1\right)}$$

$$B_{J}\left(\frac{g_{J}\mu_{B}JB}{k_{B}T}\right)=\frac{2J+1}{2J}\;\coth\left(\left(\frac{2J+1}{2J}\right)\left(\frac{g_{J}\mu_{B}JB}{k_{B}T}\right)\right)-\frac{1}{2J}\;\coth\left(\left(\frac{1}{2J}\right)\left(\frac{g_{J}\mu_{B}JB}{k_{B}T}\right)\right)$$

where *M* is the magnetic moment; *M*_s_ is saturation magnetization; *g_J_* is Landé *g*-factor; and *B_J_* is the Brillouin function. Since the CW fitting result agrees with the high-spin state of Fe^2+^, *S* and *L* should be 2. As shown in the inset of Figure 2b, the total moment from the Brillouin function fitting, *J*, is 2 and is in the range of |*L* – *S*| and *L* + *S*. The deviation in the fit may arise from how accurately the temperature captures the effects of thermal frustration. Fitting is much better as a free temperature parameter at around 3 K instead of fixing at 2 K.

Figure 3a shows the temperature-dependent specific heat of FeGe_3_O_4_. The transition feature appears around 10 K. The phonon contribution exists dominantly in FeGe_3_O_4_ when the temperatures are above 40 K. If we consider only two contributions in the specific heat, *C*_p_ = *C*_phonon_ + *C*_mag_, the data was fitted using two Debye model with the temperature ranging from 40 to 100 K. This yields θ_D1_ = 307(3) K and θ_D2_ = 887(13) K, shown in Figure 3a. The inset gives the low-temperature specific heat measurement, and there is no transition observed before 0.061 K. The magnetic specific heat *C*_m_ is obtained by subtracting the phonon contribution from the fitting. The total entropy *S*_mag_ = ∫(*C*_m_/*T*)­d*T* was calculated to be around 4.6 J/mol-K. The magnetic entropy should be *S*_mag_ = *R*  ln­(2*J* + 1). Since *J* = 2, as shown in Figure 3b, the saturation magnetic entropy is *R*  ln­(5) = 13.4 J/mol/K, and only 34% of the entropy is detected above 2 K. This indicates that FeGe_3_O_4_ should be the quantum spin liquid and there is no long-range order detected, which is not similar to other trillium compounds, such as K_2_Ni_2_(SO_4_)_3_.^32^ As shown in Figure 3c, the specific heat exhibits evolving features around 10 K with an increasing magnetic field, although no sharp peaks are observed. Given the absence of anomalies in the temperature-dependent magnetization data between 2 and 300 K, we attribute these specific heat features to the short-range magnetic order. Below 40 K, the temperature-dependent specific heat displays a broad maximum near 5 K at zero magnetic field. As the magnetic field increases, this broad peak (T* ∼ 6.5 K) diminishes, while another broader maximum (T** ∼ 11.3 K) emerges and grows more prominent, as shown in the temperature-magnetic field phase diagram in Figure 3d.

**Figure 3:**
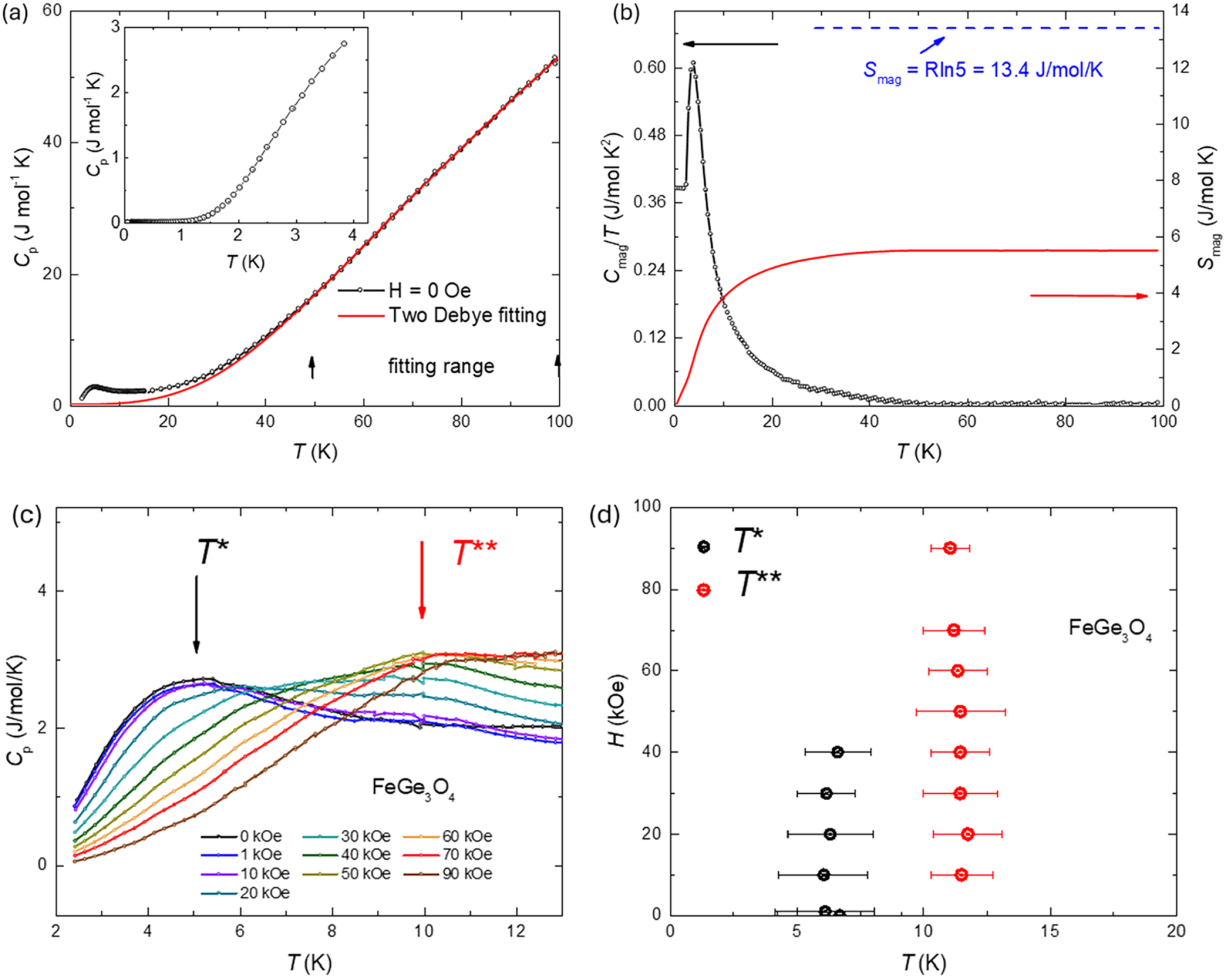
(a) Temperature-dependent total specific heat of FeGe_3_O_4_ at zero field, together with the two Debye models representing the phonon contribution. The inset gives the low-temperature specific heat measurement. (b) Zero field temperature dependence of the magnetic specific heat and magnetic entropy. (c) Temperature-dependent specific heat for various magnetic fields. T* and T** give the two peaks in the temperature-dependent specific heat. (d) Temperature-magnetic field phase diagram of FeGe_3_O_4_.

The lack of magnetic ordering is further confirmed with elastic neutron scattering. Single-crystal neutron scattering was conducted at Oak Ridge National Lab beamline 9, CORELLI. A comparison between 2 and 20 K single-crystal neutron scattering is shown in Figure 4. Between 2 and 20 K, the intensities in the low-Q range match identically within error, which is further seen from a direct subtraction (Figure 4c,d). The lack of any order seen in neutron scattering further confirms the lack of ordering seen in the previously mentioned measurements.

**Figure 4:**
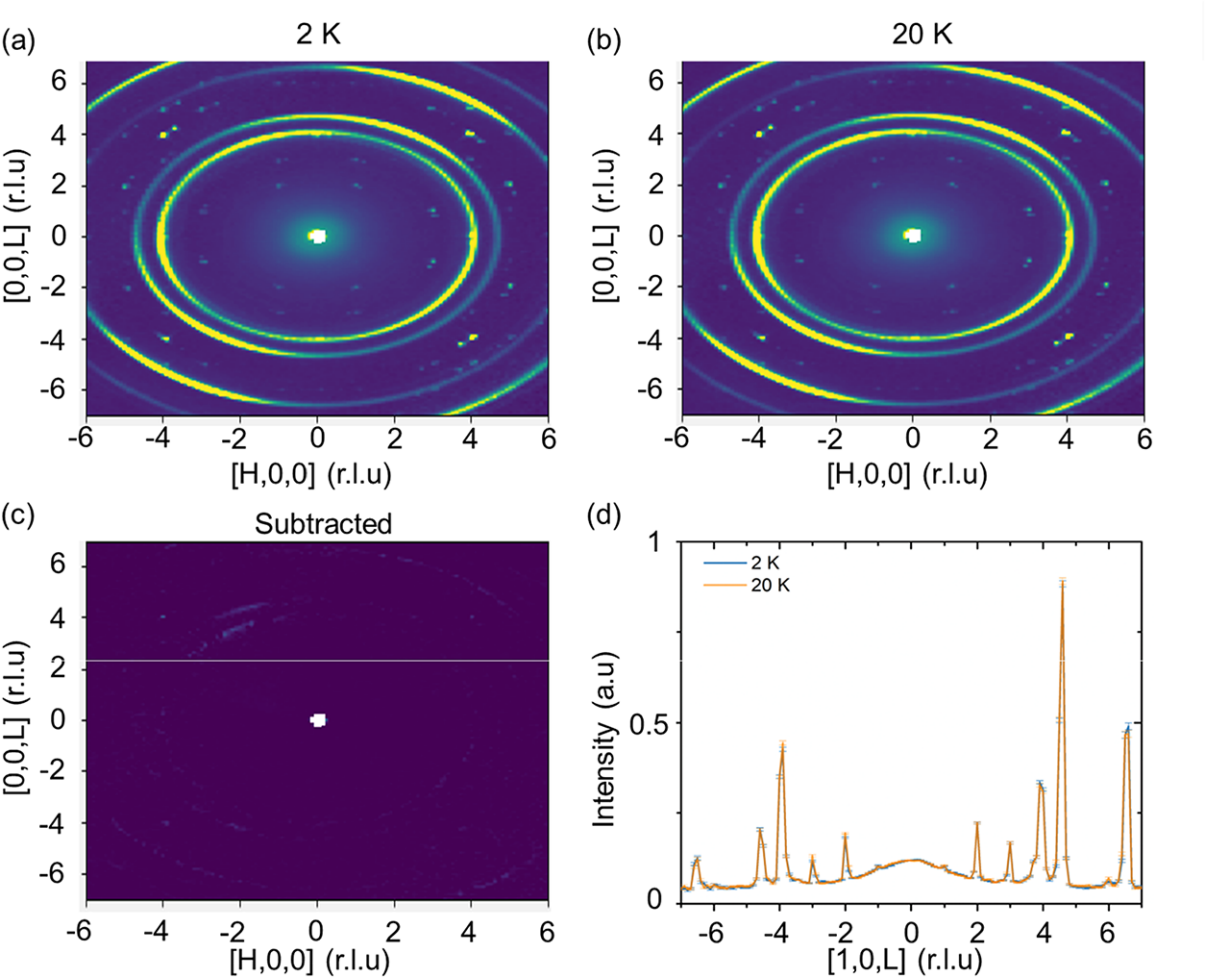
Single-crystal neutron diffraction map of FeGe_3_O_4_. (HK0) plane plots at (a) 2 K and (b) 20 K. (c) Directly subtracted map highlighting the difference between 2 and 20 K. (d) Comparative intensity plots of (10L) cut at the two temperatures.

## Conclusion

In this work, we report the synthesis and comprehensive characterization of FeGe_3_O_4_, a previously unrecognized oxide that crystallizes in the noncentrosymmetric cubic space group *P*2_1_3 and hosts an intricate double-trillium lattice of Fe atoms. Single-crystal structural refinements confirm well-defined, fully ordered Fe sites with no detectable site mixing, establishing a robust structural platform for unconventional magnetism. Magnetic susceptibility and heat-capacity measurements reveal no signatures of long-range magnetic order down to 0.06 K, despite the presence of strong local moments, which is an immediate indication of geometric frustration within the Fe sublattice. The nonlinear but nonsaturated magnetization at low temperature, together with a broad specific heat anomaly, substantial suppression of magnetic entropy, and diffuse features in neutron scattering, all point toward a short-range correlated, highly frustrated magnetic state. These combined experimental results establish FeGe_3_O_4_ as a rare oxide platform in which geometric frustration, competing exchange interactions, and noncentrosymmetric lattice symmetry converge, offering fertile ground for exploring unconventional magnetic phenomena in three-dimensional frustrated systems.

## Supplementary Material



## Notes

Crystal data and structure refinement of FeGe_3_O_4_ at 80 K; atomic coordinates and isotropic atomic displacement parameters (Å^2^); cation’s information on Fe–Ge–O systems; phase diagram of Fe–Ge–O; magnetic susceptibility and magnetic field dependence of magnetization; total and decomposition of band structure for FeGe_3_O_4_; DFT phonon band structure and total density of states for FeGe_3_O_4_; partial phonon density of states for FeGe_3_O_4_; and magnetic exchange energies (PDF)
