# Supplementary material for: Frustrated Magnetism in FeGe3O4 with a Chiral Trillium Network
Source: J Am Chem Soc. 2026 Feb 12;148(7):7731–7. doi: 10.1021/jacs.5c22025 (PMC12951449; doi:10.1021/jacs.5c22025)
Supplement: Supplementary file 1 [file ja5c22025_si_001.pdf]

Supporting Information

Frustrated Magnetism in $FeGe_3O_4$ with a Chiral Trillium Network

Matt Boswell[1#], Mingyu Xu[1#], Haozhe Wang[1], Mouyang Cheng[2,3,4], Na Li[5], Xuefeng Sun[5], Haidong Zhou[6], Huibo Cao[7], Mingda Li[2,8], Weiwei Xie[1]*

1. Department of Chemistry, Michigan State University, East Lansing, MI, 48824 USA
2. Quantum Measurement Group, Massachusetts Institute of Technology, Cambridge, MA, 02139 USA
3. Department of Materials Science and Engineering, Massachusetts Institute of Technology, Cambridge, MA, 02139 USA
4. Center for Computational Science & Engineering, Massachusetts Institute of Technology, Cambridge, MA, 02139 USA
5. Anhui Provincial Key Laboratory of Magnetic Functional Materials and Device, Institutes of Physical Science and Information Technology, Anhui University, Hefei, Anhui 230601, CN
6. Department of Physics and Astronomy, University of Tennessee, Knoxville, TN 37996, USA
7. Neutron Scattering Division, Oak Ridge National Laboratory, Oak Ridge, TN 37831, USA
8. Department of Nuclear Science and Engineering, Massachusetts Institute of Technology, Cambridge, MA, 02139 USA

Corresponding Author: Weiwei Xie (xieweiwe@msu.edu)

#M.B. and M.X. contributed equally.

Table of Content

Table S1. Crystal data and structure refinement of $FeGe_3O_4$ at 293 K……………………………S2

Table S2. Atomic coordinates and isotropic atomic displacement parameters (Å^2)……………S2

Table S3. Information on cation compositions and cation valence in the Fe-Ge-O……………S3

Fig. S1. Chemical composition phase diagram of Fe-Ge-O ternary system……………………..S4

Fig. S2. Temperature dependence of magnetic susceptibility ………………………………..S5

Fig. S3. Band structure for $FeGe_3O_4$……………………………………………………………S6

Fig. S4. Decomposition of band structure for $FeGe_3O_4$…………………………………………S7

Fig. S5. DFT phonon band structure and total density of states for $FeGe_3O_4$……………………S8

Fig. S6. Partial phonon density of states for $FeGe_3O_4$……………………………………………S9

Table S4. Magnetic exchange energies on the Heisenberg model………………………………S10

Table S1. Crystal data and structure refinement of $FeGe_3O_4$ at 293 K.

**Chemical formula**	**$FeGe_3O_4$**
Temperature	293(2) K
Formula weight	337.62 g/mol
Space group	$P2_13$
Unit cell dimensions	a = 9.40073(7) Å
Volume	830.78(2) Å^3
Z	8
Density (calculated)	5.299 g/cm^3
Absorption coefficient	24.806 mm^{-1}
$F(000)$	1232
θ range	3.064 to 41.314°
Reflections collected	34577
Independent reflections	1840 [R_{int} = 0.0158]
Refinement method	Full-matrix least-squares on F^2
Data / restraints / parameters	1840 / 0 / 50
Final R indices	R_1 (I>2σ(I)) = 0.0132; wR_2 (I>2σ(I)) = 0.0294
	R_1 (all) = 0.0145; wR_2 (all) = 0.0296
Largest diff. peak and hole	+0.469 e/Å^3 and –0.531 e/Å^3
R.M.S. deviation from mean	0.119 e/Å^3
Goodness-of-fit on F^2	1.154

Table S2. Atomic coordinates and equivalent isotropic atomic displacement parameters (Å^2) of $FeGe_3O_4$ at 80 K. U_{eq} is defined as one third of the trace of the orthogonalized U_{ij} tensor.

	**Wyck.**	***x***	***y***	***z***	**Occ.**	**U_{eq}**
**Ge1**	12*b*	0.14588(2)	0.61464(2)	0.39609(2)	1	0.00475(4)
**Ge2**	12*b*	0.10069(2)	0.36947(2)	0.14855(2)	1	0.00391(4)
**Fe1**	4*a*	0.64021(2)	0.64021(2)	0.64021(2)	1	0.00298(6)
**Fe2**	4*a*	0.38427(3)	0.38427(3)	0.38427(3)	1	0.00589(7)
**O1**	12*b*	0.00338(15)	0.20118(15)	0.43985(14)	1	0.0080(2)
**O2**	12*b*	0.19354(15)	0.46317(15)	0.28749(15)	1	0.0079(2)
**O3**	4*a*	0.19476(14)	0.19476(14)	0.19476(14)	1	0.0053(3)
**O4**	4*a*	0.01426(15)	0.01426(15)	0.01426(15)	1	0.0064(3)

Table S3. Information on cation compositions and cation valence in the Fe-Ge-O.

Compounds	Space Group	Fe	Ge	O
$\mathbf{Fe_3Ge_2O_8}$	$P2_1/c$	2+/3+	4+	-2
$\mathbf{Fe_{15}Ge_8O_{36}}$	P-1	2+/3+	4+	-2
$\mathbf{FeGeO_3}$	$C2/c$	2+	4+	-2
$\mathbf{Fe_2GeO_4}$	$Fd\text{-}3m$	2+	4+	-2
$\mathbf{FeGe_3O_4}$	$P2_13$	2+	2+	-2

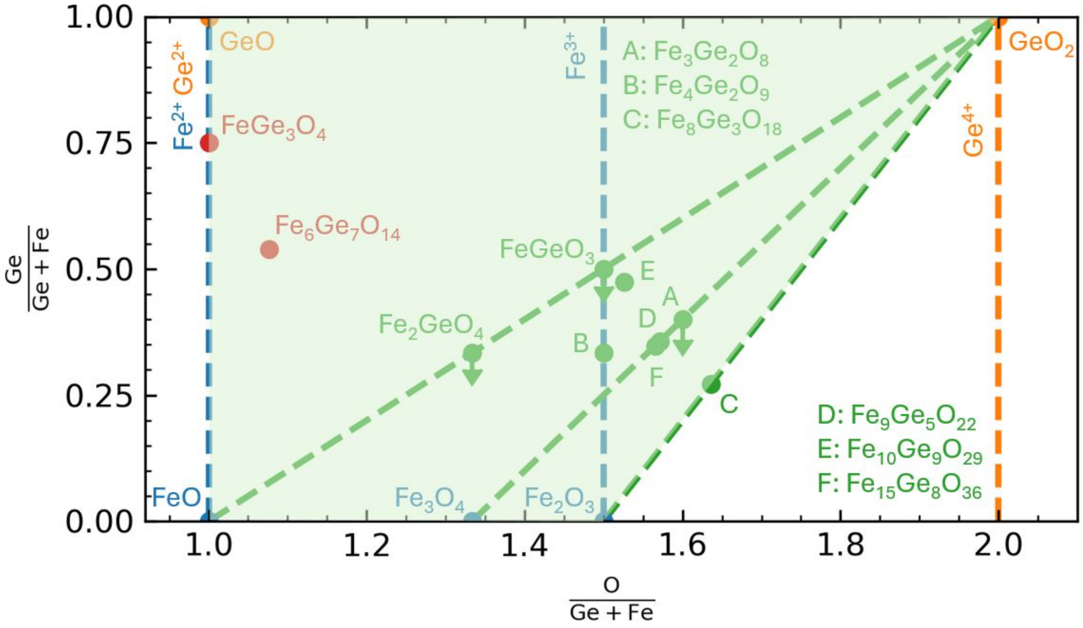

Fig. S1. Chemical composition phase diagram of Fe-Ge-O ternary system. Data points represent some of the reported Fe-Ge-O phases. Arrows indicate chemical doping (Fe or Ge) of the parent phases. The green shadow suggests the area of existing Fe-Ge-O ternary compounds.

Chemical Composition Mapping for New Oxides: Before delving into the structural and property details of $FeGe_3O_4$, we introduce a novel chemical composition map for empirically exploring new oxide materials, as illustrated in **Fig. S1**. The map utilizes the *x*-axis to represent the O/(Ge+Fe) ratio. For reference, the O/(Ge+Fe) ratio is 2 in GeO_2 and 1 in GeO, while the O/(Ge+Fe) ratios are 1.5 in Fe_2O_3, 1.33 in Fe_3O_4, and 1 in FeO. Fe typically exhibits stable oxidation states of 2+ and 3+, whereas Ge stabilizes in oxidation states of 2+ and 4+. Under mild experimental conditions, ternary Fe-Ge-O phases generally incorporate combinations of Fe^{3+}, Fe^{2+}, Ge^{2+}, and Ge^{4+}. This allows us to construct compositional lines connecting GeO_2 with Fe_2O_3, Fe_3O_4, and FeO. As anticipated, most of the reported ternary phases (highlighted in green) fall along these dashed lines. Theoretical constraints suggest that ternary phases cannot exist beyond the boundary defined by the GeO(blue)- Fe_2O_3(green) line due to the maximum oxidation states of Ge^{4+} and Fe^{3+} (The green shadow suggests the area of existing Fe-Ge-O ternary compounds.). Crossing this boundary would require highly oxidizing conditions capable of stabilizing Ge and Fe in oxidation states greater than 4+ and 3+, respectively. An intriguing example is $Fe_4Ge_2O_9$, positioned along the blue line in **Fig. S1**. This compound can be described as a mixture of Fe^{2+}/Fe^{3+} with Ge^{2+}. The new discovery $FeGe_3O_4$ compound sits on the boundary of Fe^{2+} and Ge^{2+}.

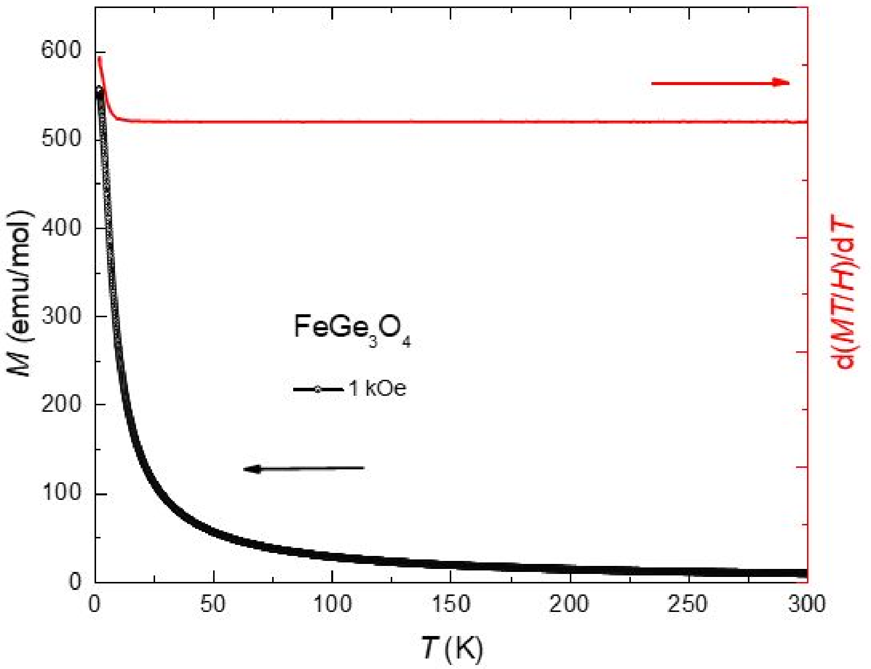

Fig. S2. Temperature dependence of magnetic susceptibility at 1000 Oe and d(MT/H)/dT as a function of temperature.

Density Functional Theory (DFT) calculations are conducted using the Vienna Ab initio Simulation Package (VASP). The projector-augmented wave (PAW) method is employed, with exchange-correlation effects described by the Perdew-Burke-Ernzerhof (PBE) formulation of the generalized gradient approximation (GGA). The plane-wave cutoff energy is set at 520 eV to ensure sufficient convergence. The system under investigation, $FeGe_3O_4$, is treated in a non-spin-polarized configuration in consistency with experimental observations. A calibrated Hubbard U correction of 5.3 eV, as used by the Materials Project, is applied to better account for the on-site Coulomb interactions in the Fe *d* orbitals. Calculations are performed with a $3 \times 3 \times 3$ *k*-point mesh centered at the Gamma point, to sample the Brillouin zone. Geometry optimization is carried out with a convergence criterion for the forces set to 0.02 eV / Å under the symmetric constraint of $P2_13$ space group. Our data analysis on DFT results utilizes the VASPKIT package, and the phonon properties are calculated combining VASP with the Phonopy package with a $2 \times 2 \times 1$ supercell.To understand the magnetic and electronic behaviors of $FeGe_3O_4$, the electronic structure was calculated and shown in **Fig. S3**. The calculated band gap is around 3 eV, indicating the insulating properties of $FeGe_3O_4$. Decomposition of band structure for $FeGe_3O_4$ shown in **Fig. S3*b*** on each of the three elements Fe, Ge and O shows that the hybridization between Fe and O atoms dominating the Fermi level. [1-7]

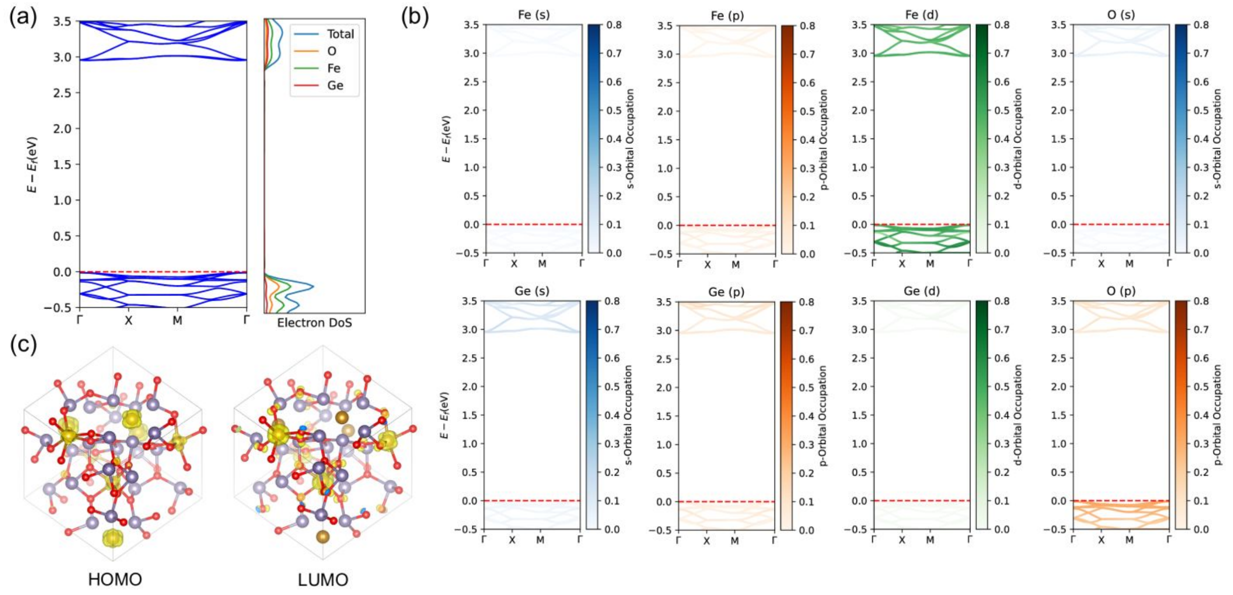

Fig. S3. Electronic structure of $FeGe_3O_4$ calculated by DFT. (*a*) Electronic band structure and projected density of states (PDOS) of the material, with contributions from O, Fe, and Ge atoms. The Fermi level is set to 0 eV (red dashed line). **(*b*)** Orbital-projected band structure, showing the contributions of Fe, Ge, and O atoms in different orbitals (s, p, d). The color intensity represents the orbital occupation. **(*c*)** Partial charge distribution of the highest occupied molecular orbital (HOMO) and the lowest unoccupied molecular orbital (LUMO).

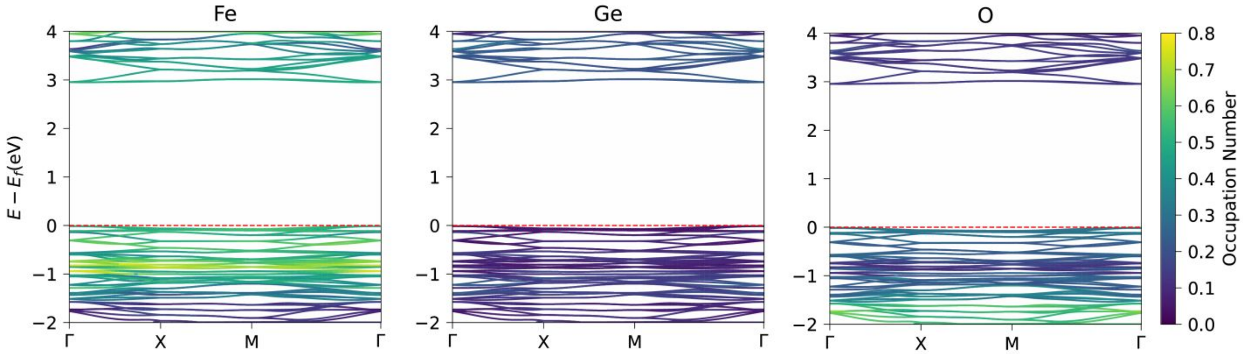

Fig. S4. Decomposition of band structure for $FeGe_3O_4$ on each of the three elements Fe, Ge and O. The occupation on each band for each element is shown with a colormap from 0 to 1.

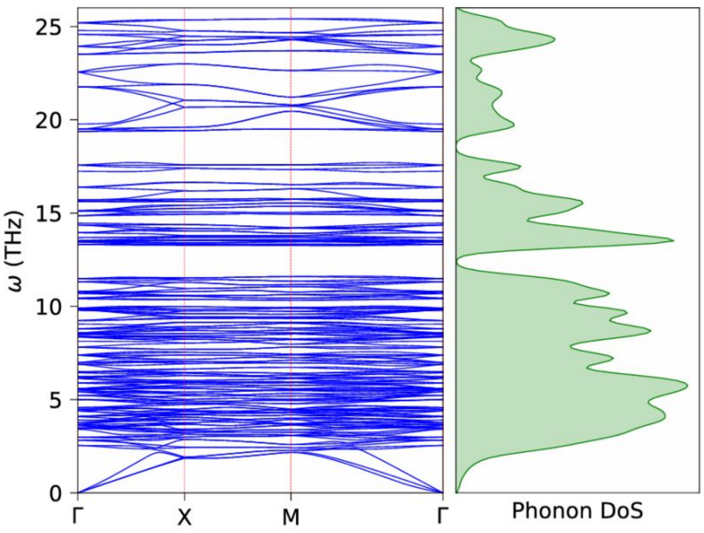

Fig. S5. DFT phonon band structure and total density of states for $FeGe_3O_4$. The band structure is plotted along the Γ-X-M-Γ symmetry path, and the phonon density of states is smeared out for smoothness.

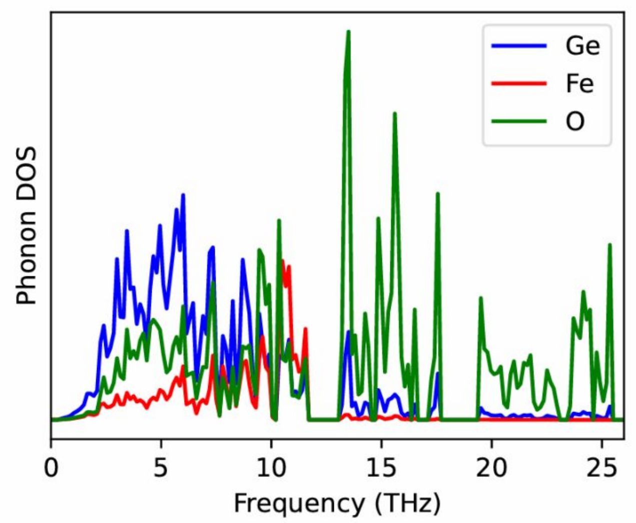

Fig. S6. Partial phonon density of states for $FeGe_3O_4$, projected on each of the three elements Ge, Fe and O.

1. Kresse, G.; Furthmüller, J. Efficient Iterative Schemes for *Ab Initio* Total-Energy Calculations Using a Plane-Wave Basis Set. *Phys Rev B* **1996**, *54* (16), 11169–11186. https://doi.org/10.1103/PhysRevB.54.11169.
2. Blöchl, P. E. Projector Augmented-Wave Method. *Phys Rev B* **1994**, *50* (24), 17953–17979. https://doi.org/10.1103/PhysRevB.50.17953.
3. Kresse, G.; Joubert, D. From Ultrasoft Pseudopotentials to the Projector Augmented-Wave Method. *Phys Rev B* **1999**, *59* (3), 1758–1775. https://doi.org/10.1103/PhysRevB.59.1758.
4. Perdew, J. P.; Burke, K.; Ernzerhof, M. Generalized Gradient Approximation Made Simple. *Phys Rev Lett* **1996**, *77* (18), 3865–3868. https://doi.org/10.1103/PhysRevLett.77.3865.
5. Jain, A.; Ong, S. P.; Hautier, G.; Chen, W.; Richards, W. D.; Dacek, S.; Cholia, S.; Gunter, D.; Skinner, D.; Ceder, G.; Persson, K. A. Commentary: The Materials Project: A Materials Genome Approach to Accelerating Materials Innovation. *APL Mater* **2013**, *1* (1). https://doi.org/10.1063/1.4812323.
6. Wang, V.; Xu, N.; Liu, J.-C.; Tang, G.; Geng, W.-T. VASPKIT: A User-Friendly Interface Facilitating High-Throughput Computing and Analysis Using VASP Code. *Comput Phys Commun* **2021**, *267*, 108033. https://doi.org/10.1016/j.cpc.2021.108033.
7. Togo, A.; Tanaka, I. First Principles Phonon Calculations in Materials Science. *Scr Mater* **2015**, *108*, 1–5. https://doi.org/10.1016/j.scriptamat.2015.07.021.

To extract the magnetic exchange parameter J, we use density functional theory (DFT) to compute the magnetic energies of a series of spin configurations for $FeGe_3O_4$. These energies are then mapped onto the Heisenberg spin Hamiltonian with nearest neighbor (NN) couplings J_1 and next nearest neighbor (NNN) couplings J_2 :

$$H = J_1 \sum_{\langle i,j \rangle} S_i \cdot S_j + J_2 \sum_{\langle\langle i,j \rangle\rangle} S_i \cdot S_j,$$

The DFT results reveal distinct magnetic behavior for the Fe atoms in the system. Among the 8 Fe atoms in the $FeGe_3O_4$ unit cell, four of them each surrounded by Ge atoms, exhibit a non-magnetic configuration (with localized spin < 0.04 μ_B) even under spin polarizations, contributing negligibly to the magnetic moment; while the remaining four Fe atoms, located in an O_h symmetry environment coordinated by six O atoms, display a high-spin configuration with magnetic moments exceeding 3.5 μ_B. This is consistent with the MH measurement. Thus, we only consider these four high-spin Fe sites as magnetic, with their spins contributing to the exchange interactions and overall magnetic properties. The calculated results for magnetic exchange energies are tabulated below, where both the nearest-neighbor (NN) coupling indicates ferromagnetic (FM) order in the short length scale. It is worth noticing that as the Hubbard U is turned on to 5.3 eV, where exchange coupling terms get strongly suppressed, the NNN coupling J_2 flips sign to a positive number, indicating anti-ferromagnetic (AFM) coupling. Moreover, the AFM coupling J_2 is nearly 1/3 of the NN FM coupling J_1, and it is very likely that the competition between J_1 and J_2 leads to the magnetic frustration of $FeGe_3O_4$, which leads to the absence of magnetic order on the long range.

Table 4. Magnetic exchange energies obtained by DFT energy mapping on the Heisenberg model, with the Hubbard U parameter set as 0 and 5.3 eV. The nearest-neighbor (NN) and next-nearest-neighbor (NNN) coupling correspond to Fe–Fe bond distances of 5.76 Å and 8.61 Å, respectively.

Ex	Fe–Fe	Bond length (Å)	U = 0 eV	U = 5.3 eV
J_1	Fe1–Fe2	4.1680(4)	/	/
J_1	Fe1–Fe2	4.1680(4)	/	/
J_2	Fe1–Fe2	5.0262(4)	/	/
J_3	Fe1–Fe2	5.4426(4)	/	/
J_4	Fe2–Fe2	5.7602(4)	-3.60 K	-0.45 K
J_4	Fe1–Fe1	5.7694(3)		
J_5	Fe1–Fe1	8.4861(4)	-1.61 K	+0.14 K
J_5	Fe2–Fe2	8.6052(5)		
